# Mental healthcare access among resettled Syrian refugees in Leipzig, Germany

**DOI:** 10.1017/gmh.2024.16

**Published:** 2024-02-06

**Authors:** Samantha F. Schoenberger, Kim Schönenberg, Daniela C. Fuhr, Yuriy Nesterko, Heide Glaesmer, Egbert Sondorp, Aniek Woodward, Marit Sijbrandij, Pim Cuijpers, Alessandro Massazza, Martin McKee, Bayard Roberts

**Affiliations:** 1 Department of Health Services Research and Policy, London School of Hygiene and Tropical Medicine, London, UK; 2 Department of Medical Psychology and Medical Sociology, University of Leipzig, Leipzig, Germany; 3 Research Group Implementation Research and Mental Health, Leibniz Institute of Prevention Research and Epidemiology, Bremen, Germany; 4 Health Sciences, University of Bremen, Bremen, Germany; 5 Research Department, Center ÜBERLEBEN, Berlin, Germany; 6 Department of Global Health, KIT Royal Tropical Institute, Amsterdam, the Netherlands; 7 Department of Clinical, Neuro and Developmental Psychology, Amsterdam Public Health Research Institute, Vrije Universiteit Amsterdam, Amsterdam, the Netherlands

**Keywords:** refugee, health services, depression, anxiety, post-traumatic stress

## Abstract

Our aim was to examine mental health needs and access to mental healthcare services among Syrian refugees in the city of Leipzig, Germany. We conducted a cross-sectional survey with Syrian refugee adults in Leipzig, Germany in 2021/2022. Outcomes included PTSD (PCL-5), depression (PHQ-9), anxiety (GAD-7) and somatic symptom (SSS-8). Descriptive, regression and effect modification analyses assessed associations between selected predictor variables and mental health service access. The sampling strategy means findings are applicable only to Syrian refugees in Leipzig. Of the 513 respondents, 18.3% had moderate/severe anxiety symptoms, 28.7% had moderate/severe depression symptoms, and 25.3% had PTSD symptoms. A total of 52.8% reported past year mental health problems, and 48.9% of those participants sought care for these problems. The most common reasons for not accessing mental healthcare services were wanting to handle the problem themselves and uncertainty about where to access services. Adjusted Poisson regression models (*n* = 259) found significant associations between current mental health symptoms and mental healthcare service access (RR: 1.47, 95% CI: 1.02–2.15, *p* = 0.041) but significance levels were not reached between somatization and trust in physicians with mental healthcare service access. Syrian refugees in Leipzig likely experience high unmet mental health needs. Community-based interventions for refugee mental health and de-stigmatization activities are needed to address these unmet needs in Leipzig.

## Impact statement

Our study with Syrian refugees in Leipzig, Germany showed a high burden of mental health distress and commonly reported exposure to traumatic events and discrimination. Around half of respondents self-reported having a mental health or emotional problem but had not sought any sources of care or support. Key barriers to accessing support were not knowing where to seek care, concern about language barriers with health care barriers, stigma around mental health, and fear of discrimination. For those who did seek some kind of care, members of their community were the first source of support. These findings provide new evidence supporting the need for community-based mental health and psychosocial support services for Syrian refugees in Leipzig. However, such services remain limited in Leipzig and there is a need for different types of services that may be more relevant, accessible, and scalable for Syrian refugees. This strengthens arguments for task-shifting of mental health services from licensed providers to trained lay health care providers from within Syrian refugee communities in Leipzig. We also observed high levels of somatization. This suggests that health professionals treating refugee populations in Leipzig could benefit from training on the ways that mental distress can manifest physically, and ways to educate patients about these manifestations. Community-level activities could also be conducted to raise awareness of the ways that trauma and mental distress can be physically felt and manifested. In summary, the study findings reiterate the need for improved access to mental health and psychosocial support for the Syrian refugee community in Leipzig.

## Introduction

Refugees are at increased risk of mental ill health as they are exposed to adverse situations before, during, and after displacement and arrival to host countries (Miller and Rasmussen, 2016; Blackmore et al., 2020). These include forced displacement, violence, unsafe transport, the ongoing stress of insecure housing, unemployment or precarious employment, poverty, food insecurity, discrimination, isolation and loss of social support. Research undertaken in countries receiving Syrian refugees places mental health among their most pressing needs (El Arnaout et al., 2019), yet refugees consistently face barriers to obtaining mental healthcare, due to difficulty navigating health systems after resettlement, the stigma associated with mental illness, language skills, and low levels of trust in physicians and formal care systems (Cheng et al., 2015; Kotovicz et al., 2018; Satinsky et al., 2019; Hendrickx et al., 2020; van der Boor and White, 2020).

Germany hosts 1.2 million refugees, including the largest group of Syrian asylum seekers in Europe, first arriving in large numbers in 2011. These Syrian refugees were resettled in every German state, in numbers proportional to state population size. The number of Syrians in Leipzig (the location of our study), grew from 687 in 2012 to 10,709 in 2021 to become the largest single group of migrants in the city (ASW (Leipzig), 2022). By 2020, approximately 97% of Syrians lived in residential accommodation, with 3% in refugee facilities. The German Asylum Seekers’ Benefits Act (“Asylbewerberleistungsgesetz”) provides basic support for food, housing, clothing and healthcare to those awaiting legal confirmation of refugee status, including subsidies for residential accommodation after 18 months of residence. Once they receive this confirmation, they qualify for the social benefits available to the rest of the German population and can enter the job market, while they must also pledge to participate in integration and language classes. Nevertheless, Syrians in Leipzig still experience high levels of poverty. The average net equivalent income in the Syrian population in Leipzig in 2020 was 800€ per household, around half the income compared to the German general population in Leipzig. A total of 63% of Syrians in Leipzig were at risk of poverty (compared to 16% in the German general population in Leipzig) and 26% were unemployed (compared to 4% in the German general population) in 2020 (Stadt Leipzig, 2020).

Although the Asylum Seekers’ Benefits Act entitles asylum seekers to basic healthcare, this is primarily to enable them to access healthcare for acute medical conditions (Hyde, 2016; Ossege and Köhler, 2016). Usually, full access is granted 18 months after arrival in Germany. However, some federal states in Germany, but not Saxony where this study was conducted, provide a health insurance card conferring full healthcare access on arrival in Germany.

Refugees without a health insurance card can only access mental healthcare services beyond acute crisis interventions on a case-by-case basis following approval by the regional council or social welfare office of any referrals to an outpatient physician, including psychiatrists or counselors (Offe et al., 2018; Nikendei et al., 2019). This risks late diagnosis of mental disorders for those unfamiliar with the system, which can exacerbate symptom severity and functional impairment (Bauhoff and Göpffarth, 2018). Mental health care services established specifically for refugees are administered through Psychosocial Care Centers for Refugees and Torture Survivors, with services including psychosocial counseling, psychotherapy, mental health assessment, medical documentation of torture, legal and social support while undergoing the asylum procedure, and other services such as assistance in finding appropriate housing. However, demand for these services far exceeds capacity, and the average waiting time for admission is over 6 months, with many potential clients rejected entirely (Mohammed and Karato, 2022). Once they are granted asylum, refugees are entitled to the same health services available to German citizens (Elsouhag et al., 2015; Horlings and Hein, 2018), but interpretation services are not covered (Ossege and Köhler, 2016).

There is a high burden of mental health needs among Syrian refugees in Germany. While the prevalence of mental health symptoms can vary substantially, depending on the population concerned and the instruments used, surveys among refugees typically report that 35–53.3% experience anxiety, 21.7–57.1% depression, and 13–34.9% PTSD (Führer et al., 2016; Georgiadou et al., 2017; Biddle et al., 2019; Nesterko et al., 2020a). High rates of somatic distress have also been reported among Syrian refugees in Germany, with implications for healthcare providers in recognizing the mental health needs underlying these reported physical symptoms (Nesterko et al., 2020b; Renner et al., 2020; Zbidat et al., 2020; Borho et al., 2021). However, research into the facilitators and barriers for Syrian refugees accessing mental health services in Germany remains quite limited, including the predictors of mental healthcare access (Nesterko et al., 2020a; Zbidat et al., 2020).

Our study involved adult Syrian refugees from the city of Leipzig in the State of Saxony, in eastern Germany, and was in response to a need expressed by stakeholders for more evidence on the mental health burden and patterns of access to mental health services among this population. The aim of our study was to assess the mental health needs of Syrian refugees in Leipzig, Germany, and their access to mental healthcare services. Our specific objectives were to: (1) describe the prevalence of symptoms of key mental disorders; (2) examine levels of mental health care access and barriers to care; (3) determine predictors of mental health care access by Syrian refugees and (4) understand how access is modified by the effect of current mental health symptomology on care-seeking behavior.

## Methods

We collected data as part of the Syrian REfuGees MeNTal HealTH Care Systems (STRENGTHS) research consortium, which implemented and evaluated scalable psychological interventions for Syrian refugees in Europe and the Middle East (Sijbrandij et al., 2017; Graaff et al., 2022).

### Study design, population and sampling

We conducted a cross-sectional postal survey between September 2021 and March 2022. We had intended to conduct it face-to-face but had to send it by post due to the COVID-19 pandemic. Our sampling frame was an official list of all adult Syrian migrants (aged +18 years) registered with the Leipzig municipality. The target sample size was *n* = 500 (see Supplementary Material 1 for sample size calculation). We had to use two waves of sampling to achieve this number. The first wave of 3,001 survey invitations was dispatched on September 6, 2021, with adverts also placed on public transport and in locations used by the Syrian community (such as supermarkets and cultural centers) to encourage uptake by those that had received the postal survey. To try and gather representative data in the first wave, the sampling frame was stratified by age, gender, and postcode. As we did not achieve the desired sample size in the first wave (including after reminders were sent after 6 weeks), a second booster wave of invitations was sent to a further 2,861 people on January 20, 2022 (with reminders sent after 6 weeks). Taking waves 1 and 2 together, all Syrian adults registered in the sampling frame were contacted. In the first wave, there was a 14.2% initial response rate, and the second wave had a 9.36% initial response rate (the average initial response rate across the two waves was 11.9%). The final response rate was 9.6% (*n* = 513) for those who provided written consent and participated in the survey. Details of ethics procedures are provided at the end of the manuscript.

### Survey questionnaire

We developed a questionnaire covering the following domains: (i) sociodemographic characteristics (age, sex, educational level, marital status, parenthood, household economic situation, employment status; (ii) migration characteristics and levels of integration into German society (arrival date in Leipzig, integration into Germany, experience of discrimination, and German language skill using a single item self-reported measure worded as “In your opinion, how well are your German language skills?” with the response option a range from 0 to 10 and then categorized in four categories from none to excellent (El Khoury, 2019); (iii) general health status; (iv) exposure to traumatic events using the DSM-5 Life Events Checklist (LEC-5); (v) trust in physicians using the Wake Forest Trust in Physicians Scale; (vi) access to mental health care using a self-reported “yes/no” response to the question “In the past year, have you sought care for feelings such as anxiety, nervousness, being restless, tiredness, difficulty with sleeping or any other emotional or behavioral problems?”; and then reasons for not seeking health care; and (vii) mental health status. Mental health status was assessed using the Patient Health Questionnaire-9 (PHQ-9) for moderate/severe depression in the past 2 weeks (PHQ-9 score ≥10), the Generalized Anxiety Disorder Scale (GAD-7) for moderate/severe anxiety in the past 2 weeks (GAD-7 score ≥10), and the Posttraumatic Stress Disorder Checklist for DSM-5 (PCL-5) for PTSD in the past 4 weeks (PCL-5 score ≥33). We also produced a summary binary mental health symptomology variable with participants who scored “moderate/severe depression symptoms,” and/or “PTSD symptoms,” and/or “moderate/severe anxiety symptoms.” Other health outcome measures included the Somatic Symptom Scale-8 (SSS-8) (past 7 days). Further details on the measures are in Supplementary Material 2. All study materials were translated from English to Arabic and German and independently back-translated and then piloted following standard procedures. Arabic versions of the PHQ-9, GAD-7, SSS-8, LEC-5, and PCL-5 have previously been validated, including with Syrian refugees in other settings (Fuhr et al., 2020).

### Data analysis

To examine factors associated with access to mental health services, we focused on three predictor variables: (a) the summary measure of current mental health symptoms (see above); (b) current somatization (measured by the SSS-8); and (c) “trust in physicians” (Wake Forest Trust in Physicians Scale). Current mental health symptoms were chosen as a potential predictor because of their relevance to psychosocial intervention development. If regression analyses find that this is not a predictor of mental healthcare service access, mental health actors may need to consider how to better engage those in distress than current strategies. Current somatization was selected based on previous research into cross-cultural differences in the manifestation of mental ill health and the suggestion that Syrian populations may not seek support until ill health manifests in the form of physical distress (Nesterko et al., 2020a; Renner et al., 2020; Zbidat et al., 2020; Borho et al., 2021). Trust in physicians was selected because of its influence on mental health care-seeking behavior (Cheng et al., 2015; Kotovicz et al., 2018).

In our initial exploration of the survey data, we observed that the outcome variable (mental health service access) did not follow a normal distribution, thus generalized linear models were used in which response variables follow distributions that are not normal. Led by previous conventions for fitting Poisson regression models for skewed binary outcome variables, a series of three Poisson models were built to assess associations between each predictor (recent mental health symptoms, somatization, and trust in physicians) and the outcome of interest (access to mental healthcare services). First, a crude baseline model was created using each predictor variable using Fisher’s for categorical variables and Chi-square for binary variables. This analysis did not find significant associations between gender, marital status, or employment status and access to mental healthcare services, and so these variables were excluded. Generalized linear Poisson regression models were used separately for each predictor. We then built a list of *a priori* and hypothesized confounding variables for each model, based on initial testing of bivariate associations between predictor and outcome variables, previous empirical research, and our judgment. We identified important confounders in the final adjusted model by comparing the crude and adjusted risk ratio (RR) with and without each variable in the expanded and nested regression models. Given the constraints of the sample size, we included only those two or three confounders that most changed the RR in the final model. Final models were compared with unadjusted models using Akaike information criterion (AIC) calculations and assessed for equidispersion (variance=mean assumption of Poisson regression models) (Zou, 2004; Armstrong-Hough et al., 2018). To test for the presence of effect modification, we conducted a stratified analysis for levels of third variables of interest. We calculated contingency tables using the epiR statistical package to compare Odds Ratios (ORs) between levels of third variables in the association between mental health symptoms and access to mental healthcare. We used the R statistical package (R version 4.2.1). Significance in all analyses was determined at *p* ≤ 0.05. For descriptive data analysis, missing data were removed. For regression and effect modification analyses, a single imputation was used to ascribe a range of sample means for numeric variables when data were missing for items on the somatization and trust in physicians scales (if they also had complete data for the outcome variable). The reliability and validity of all scales used in regression and effect modification models were assessed using Cronbach’s *α* (*α* > 0.80 “good,” *α* > 0.70 “acceptable”).

## Results

Table 1 shows the demographics and clinical characteristics of sample participants (*n* = 513). Most were male (61.0%), under 45 years old (76.8%), reported their economic situation as average (58.8%), and had attained a post-secondary education (51.4%). Just over one-third of the sample was employed (39.7%), six in 10 were married (60.7%) and had children (58.4%). While just over half of the sample had arrived in Leipzig in the last 5 years (54.8%), nearly all reported possessing a health insurance card (96.6%) which gives them full access to all health care services, and about half of the sample self-reported “good” or “excellent” German language proficiency (46.3%). Reported experiences of discrimination varied widely across settings, with 73.7% reporting it within the housing market, 70.2% in public places, and around 50% reporting it at school or work (51.9%), by authority (53.7%) and when applying for jobs (54.5%). Around one-third of participants (31.0%) reported discrimination in the healthcare system. Experience of trauma during conflict and displacement was common. Nearly two-thirds of participants (64.2%) had witnessed and/or experienced a fire or explosion and nearly 60% of the sample reported witnessing and/or experiencing severe human suffering (59.4%) and combat or exposure to a warzone (58.9%). In addition, 10.9% indicated experiencing and/or witnessing sexual assault and 30.4% reported witnessing sudden violent death (Table 2).

**Table 1.** Demographics and clinical characteristics, by sex (*n* = 513)^a^

	Total *N* (%)	Male *N* (%)	Female *N* (%)
	*N* = 513	*N* = 313 (61.0)	*N* = 200 (39.0)
Age			
18–30 years old	200 (39.0)	116 (37.1)	84 (42.0)
31–45 years old	194 (37.8)	123 (39.3)	71 (35.5)
46–95 years old	119 (23.2)	74 (23.6)	45 (22.5)
Marital status			
Single (never married)	154 (30.3)	116 (37.3)	38 (19.2)
Married/cohabiting	309 (60.7)	169 (54.3)	140 (70.7)
Divorced/separated	36 (7.1)	24 (7.7)	12 (6.1)
Widow/widower	10 (1.9)	2 (0.6)	8 (4.0)
Children			
Yes	297 (58.4)	156 (50.2)	141 (71.2)
Years of education			
Basic education (up to 9 years)	133 (26.8)	81 (26.7)	52 (26.9)
Secondary education (9–12 years)	108 (21.8)	67 (22.1)	41 (21.2)
Post-secondary education (>12 years)	255 (51.4)	155 (51.2)	100 (51.8)
Household economic situation			
Good	66 (13.7)	35 (12.0)	31 (16.4)
Average	283 (58.8)	163 (55.8)	120 (63.5)
Bad	132 (27.4)	94 (32.2)	38 (20.1)
Work situation			
Employed	185 (39.7)	153 (54.3)	32 (17.4)
Unemployed/retired	102 (21.9)	70 (24.8)	32 (17.4)
Student/retraining program	98 (21.0)	47 (16.7)	51 (27.7)
Housewife	69 (14.8)	5 (1.8)	64 (34.8)
Other	12 (2.6)	7 (2.5)	5 (2.7)
Arrival in Leipzig			
2016–2022	271 (54.8)	155 (51.0)	116 (60.7)
Before 2016	224 (45.2)	149 (49.0)	75 (39.3)
German language skill			
No German language skills	26 (5.3)	10 (3.3)	16 (8.4)
Poor German language skills	89 (18.1)	50 (16.6)	39 (20.5)
Fair German language skills	149 (30.3)	93 (30.8)	56 (29.5)
Good German language skills	138 (28.0)	82 (27.2)	56 (29.5)
Excellent German language skills	90 (18.3)	67 (22.2)	23 (12.1)
Integration^b^			
Mean (SD)	62.24 (28.34)	66.81 (26.61)	55.03 (29.54)
Discrimination			
Discrimination at school or work	244 (51.9)	157 (54.7)	87 (47.5)
Discrimination by authority	254 (53.7)	168 (57.7)	86 (47.3)
Discrimination in job searching	242 (54.5)	158 (57.0)	84 (50.3)
Discrimination in public places	342 (70.2)	207 (69.7)	135 (71.1)
Discrimination when looking for an apartment	348 (73.7)	229 (78.7)	119 (65.7)
Discrimination in healthcare	148 (31.0)	85 (29.3)	63 (33.7)
In possession of a health insurance card			
Yes	484 (96.6)	299 (97.4)	185 (95.4)
Chronic condition			
Yes	174 (40.1)	103 (39.2)	71 (41.5)
Traumatic event happened			
Yes	321 (74.3)	206 (77.7)	115 (68.9)
Traumatic event witnessed			
Yes	296 (77.1)	188 (80.0)	108 (72.5)
No	88 (22.9)	47 (20.0)	41 (27.5)
Somatization^c^			
Yes	130 (25.3)	69 (22.0)	61 (30.5)
Trust in physicians			
Mean (SD)	25.22 (6.77)	24.93 (6.89)	25.68 (6.57)
Depression symptoms^d^			
Yes	123 (28.7)	69 (26.4)	54 (32.1)
Anxiety symptoms^e^			
Mild	120 (27.1)	67 (25.1)	53 (30.1)
Moderate	53 (12.0)	31 (11.6)	22 (12.5)
Severe	28 (6.3)	16 (6.0)	(12 (6.8)
PTSD symptoms^f^			
Yes	106 (25.3)	65 (25.7)	41 (24.7)
Ever admitted to inpatient services for mental health concerns			
Yes	48 (10.6)	28 (10.1)	20 (11.3)
Ever diagnosed with mental health condition			
Yes	46 (10.2)	28 (10.1)	18 (10.2)

a Totals per item reflect the number of participants with complete data. In some cases, this was less than *n* = 513.

b Integration was assessed with the following question: “How integrated do you feel in Germany?” with a sliding 0–100 scale.

c Calculated with the SSS-8 using a cut-off score of >11.

d Calculated with the PHQ-9 using a cut-off score of >10.

e Calculated with the GAD-7 using the following cut-off scores: “≥5 mild,” “≥10 moderate” and “≥15 severe.”

f Calculated with the PCL-5 using a cut-off score of ≥33.

**Table 2.** Traumatic events experienced and/or witnessed (*N* = 513)

Event	Experienced and/or witnessed *N* (%) ^a^
Natural disasters (e.g., flood, hurricane, tornado, earthquake)	102 (20.9)
Fire or explosion	310 (64.2)
Transportation accidents (e.g., car accident, boat accident, train wreck, plane crash)	252 (51.7)
Serious accident at work, home or during recreational activity	168 (34.9)
Exposure to toxic substances (e.g., dangerous chemicals, radiation)	50 (10.3)
Physical assault (e.g., being attacked, hit, slapped, kicked, beaten up)	201 (41.5)
Assault with a weapon (e.g., being shot, stabbed, threatened with a knife, gun, bomb)	147 (32.7)
Sexual assault (rape, attempted rape, made to perform any type of sexual act through force or threat of harm)	53 (10.9)
Other unwanted or uncomfortable sexual experiences	>51 (10.5)
Combat or exposure to a warzone (in the military or as a civilian)	287 (58.9)
Captivity (e.g., being kidnapped, abducted, held hostage, prisoner of war)	133 (27.5)
Life-threatening illness or injury	118 (24.4)
Severe human suffering	281 (59.4)
Sudden violent death (e.g., homicide, suicide)	117 (26.7)
Sudden accidental death	133 (30.4)
Serious injury, harm or death you caused to someone else	47 (9.7)
Any other very stressful event or experience^b^	219 (45.7)

*Note*: Items from the LEC-5.

a *N* for each question is as follows: 487, 483, 487, 482, 484, 484, 450, 485, 484, 487, 484, 484, 473, 439, 438, 485 and 479.

b This is a standard item included in LEC-5, and further information is not available on the nature of these events/experiences.

Turning to mental health outcomes, 28.7% of participants reported depression symptoms, 18.3% reported moderate or severe anxiety symptoms, and 25.3% reported PTSD symptoms. A total of 60% of participants met the criteria for mental health symptomology using the combined summary measure. In total, 25.3% reported somatization.

**Figure 1.**
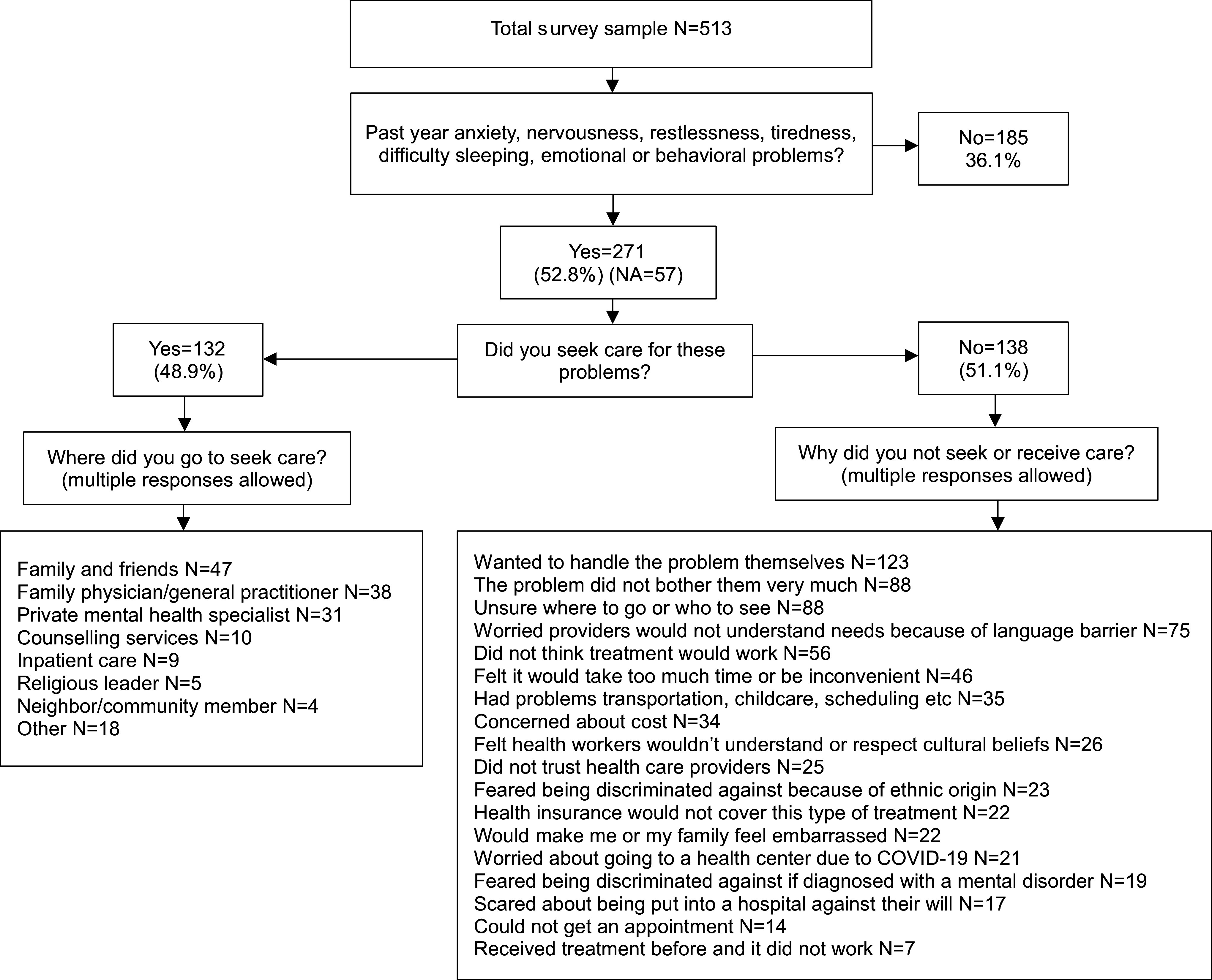
Flowchart of mental healthcare-seeking behavior (*N* = 513).

Figure 1 shows the cascade of healthcare-seeking behavior by participants. Among the total sample of 513, 271 (52.8%) reported experiencing anxiety, nervousness, restlessness, tiredness, difficulty sleeping and emotional or behavioral problems. Of them, 132 (48.9%) had sought care for mental health care concerns while 138 (51.1%) had not sought care. Among those who sought care, family and friends were the most common resource sought (*n* = 47), followed by a family physician or GP (*n* = 38) and a private mental health specialist (*n* = 31). Among those who did not seek or receive care (*N* = 138), the most common reasons were that they wanted to handle the problem on their own (*n* = 123), were unsure about where to go or who to see (*n* = 88), or the problem did not bother them too much (*n* = 88). Seventy-five participants expressed concern that providers would not understand their mental health needs due to the language barrier.

A total of 429 participants responded to the question about where people in their community first seek mental health care (Table 3). Among them, 40.3% identified family and friends as the first point of contact for people in their community, followed by family physicians (*n* = 141; 32.9%), and private mental health specialists (*n* = 118; 27.5%). Sixty-six participants (15.4%) did not know of any available mental health services in their area.

**Table 3.** Participant responses to “Where do people in your community first go to seek services for mental illness?” (*n* = 429^a^)

	Yes *N* (%)	No *N* (%)
No care available	66 (15.4)	363 (84.6)
Family physician	141 (32.9)	288 (67.1)
Counseling services	21 (4.9)	408 (95.1)
Private mental health specialists	118 (27.5)	311 (72.5)
Inpatient care	16 (3.7)	413 (96.3)
Family and friends	173 (40.3)	256 (59.7)
Neighbor/community	33 (7.7)	396 (92.3)
Religious leader	25 (5.8)	404 (94.2)
Other	21 (4.9)	408 (95.1)

*Note:* Multiple responses allowed.

a *n* = 84 “NA” responses removed.

In the regression analyses, of the 271 participants who reported mental health distress in the past year, regression models were built for participants with complete data for predictor and outcome variables (including those with calculated single imputation) (Table 4). Inclusion of age and household economic situation both led to a difference in the adjusted and unadjusted RRs for the exposure variables of mental health concerns in the past month and trust in physicians with the outcome of interest (accessing mental health services), suggesting the presence of confounding (see Supplementary Material 3). Experience of current mental health symptoms increased the likelihood of accessing mental health services by almost half RR = 1.47 (95% CI: 1.02–2.15, *P* = 0.041) in the adjusted Poisson model (age and household economic situation adjusted). There was no evidence of overdispersion. When compared to the crude model, the adjusted model improved AIC by 18.68 (433.81 vs. 415.13), indicating improved fit.

**Table 4.** Adjusted and unadjusted Poisson regression models assessing associations between predictors (current mental health condition, trust in physicians, somatization) and outcome variable (mental healthcare service access)

	Unadjusted IRR		Adjusted IRR	
	IRR (95% CI)	*p*-value	IRR (95% CI)	*p*-value
Current mental health condition^a^				
No	Reference			
Yes	1.51 (1.06–2.17)	0.024	1.47 (1.02–2.15)	0.041
Trust in physicians^b^				
No	Reference			
Yes	1.03 (0.73–1.46)	0.875	0.94 (0.65–1.36)	0.733
Somatization^c^				
No	Reference			
Yes	1.40 (0.99–1.99)	0.057	1.44 (0.99–2.10)	0.055

a Adjusted model (age and household economic status) *n* = 244.

b Adjusted model (education and household economic status) *n* = 244.

c Adjusted model (age, household economic situation and arrival in Leipzig) *n* = 240.

We asked whether participants who had greater than average trust in physicians (>25.22 for the current sample) had an increased likelihood of accessing mental health services. Including household economic situation and education both changed the RR in our crude model, again suggesting the presence of confounding. In the Poisson model that adjusted for these two variables, the RR was a nonsignificant 0.94 (95% CI: 0.65–1.36, *P* = 0.733), with no evidence for overdispersion. When compared to the crude model, the adjusted model improved AIC by 35.5 (438.99 vs. 403.49), suggesting improved fit.

Looking at somatization, adjustment for age, household economic situation, and time since arrival in Leipzig all changed the RR, suggesting the presence of confounding. In the Poisson model that adjusted for these variables, the RR for mental healthcare access in those describing somatization was 1.44 (95% CI: 0.99–2.10, *P* = 0.055), and so not reaching statistical significance. Dispersion testing found no evidence of overdispersion. Effect modification was assessed in stratified analyses, but no significant results were found (see Supplementary Material 4).

## Discussion

Our study of mental health symptoms and access to services showed that among this sample of Syrian refugees in Leipzig, participants had a high burden of mental health distress and commonly reported exposure to traumatic events and discrimination. Approximately half of respondents self-reported having a mental health or emotional problem but had not sought any sources of care or support. Mental health symptoms predicted access to mental health services. The sampling strategy means the following discussion of these findings is applicable only to Syrian refugees in Leipzig and caution should also be noted given the low response rate.

The Syrian adults in this sample reported a high burden of depression, anxiety, and PTSD symptoms, consistent with previous studies of Syrian refugee populations in other resettlement countries (Tinghög et al., 2017; Acarturk et al., 2018; Hendrickx et al., 2020; Acarturk et al., 2021). While it is not possible to make direct comparisons between these rates and those reported in our study sample, our findings do support previous evidence of a higher burden of psychological distress among populations that have experienced displacement compared to the general public in Germany (Blackmore et al., 2020).

The study showed high levels of somatic distress among the survey respondents. A study of Syrian refugees in Istanbul reported over 40% of respondents experienced moderate or severe somatization (McGrath et al., 2020). Studies report that in Syrian culture psychological distress and trauma are expressed through physical concerns such as aches, fatigue and stomach cramps (Hassan et al., 2015). Mental health concerns can be highly stigmatized in Arab cultures and seen as a weakness of character (Fakhr El-Islam, 2008), with physical distress considered more legitimate and worthy of care-seeking (Okasha, 2019). Such findings also reflect mental health difficulties among other groups of refugees and migrants from countries where mental health literacy is relatively limited. Physicians and medical professionals treating refugee populations should receive training on somatization and the ways that mental distress can manifest physically, and ways to educate patients about these manifestations. At the community level, similar education should be developed to illustrate the myriad ways that trauma and stress can be embodied (Langlois et al., 2016). Incorporating explanatory models of mental illness into treatment provision can help strengthen care plans (Hassan et al., 2015). However, it should also be recognized that somatic symptoms as measured in the study could also have been directly caused by traumatic events experienced by respondents and so might not be related to mental health problems (e.g., experiencing of toxic substances, illness, sexual assault and accidents could cause back pain, headache, pain in arms/legs without being related to mental health).

About half of the study participants self-reported some kind of mental health or emotional problem, and that they had not sought or received any care for it. The rates of mental healthcare access reported in the current study are higher than those reported in a previous study of refugees in Germany (Schlechter et al., 2021). They are also higher than rates reported by Syrian refugees resettled in other countries (Satinsky et al., 2019; Hendrickx et al., 2020). Caution is required when comparing rates across these samples, as variation across studies likely reflects differences in methodology and measurement, including the measurement of mental healthcare needs and what service types are considered in the “mental healthcare service” construct. For example, some studies may count family and friends as a source of mental healthcare (Bhui et al., 2012; Fuhr et al., 2020), while others only include encounters with health professionals. Thus, the higher rate reported in this study may be because participants identified family and friends as their first source of mental health support, while other studies might not consider these as a mental health care support.

We found that Syrian refugees experiencing mental health symptoms overwhelmingly identified family and friends as the first place to go when faced with mental illness. This is consistent with previous research finding that fellow refugees can provide a buffering effect against mental ill health and promote positive coping strategies (Khan and Hasan, 2016; Alfadhli and Drury, 2018; Hanley et al., 2018; Liamputtong and Kurban, 2018).

There were 123 respondents with mental health symptoms who preferred to handle problems themselves. This is a frequently cited reason in the mental health service literature for not seeking help, including with refugees (Mojtabai et al., 2016; Fuhr et al., 2020). Potential explanations may be that they prefer other sources of support, such as religious services. It may also reflect lower mental health literacy, mental health stigma, lower trust in mental health services, and prioritizing other needs (e.g., work, other health issues). Eighty-eight persons with mental health symptoms reported not being bothered very much, and this could potentially be attributed to diagnostic measures not being perfect. It could also be that they may have problems, but they do not experience or feel them as such. They may also feel that any problems will recede naturally. It could also reflect low mental health literacy (Mojtabai et al., 2016).

Refugees participating in our study also reported not knowing where to go to seek mental health support in Leipzig and a fear that providers would not understand their needs because of language barriers. This is a reminder of the need for linguistic and cultural adaptation to the needs of Syrian refugees engaging with the healthcare system in Leipzig. However, research suggests such services are often unavailable (Böttche et al., 2016), and there is a need for different types of services that may be more relevant, accessible and scalable (Sijbrandij et al., 2017). For example, there is evidence to support task-shifting of scalable mental health services from licensed providers to lay health care providers who are trained to deliver counseling and psychoeducation (but not psychotherapy per se) within their communities, including for refugees, and this may be an important means of increasing access to mental healthcare (Bryant et al., 2022; de Graaff et al., 2023; Schafer et al., 2023). In addition, health workers need to be trained in providing culturally relevant services, and when services are not available in a patient’s preferred language, interpretation resources are provided (Woodward et al., 2023). A survey found that over half of German healthcare professionals reported needing better training in PTSD and other mental health issues for their work with refugees (Nijman et al., 2021). Given that general practitioners were cited as a common source of support for mental health concerns, there is a need to provide training for German health workers about the mental health needs of refugees and how they can help patients navigate these services. As for specialists, while German psychotherapists report a willingness to treat refugees, they have previously reported concerns about the unavailability of translators, differing expectations of psychotherapeutic services, and communication barriers (Asfaw et al., 2020). Studies have also documented significant waiting periods for specialist mental healthcare services and the challenges faced by refugees trying to navigate the health system (Mewes et al., 2016).

The Syrian refugees in this sample reported experiencing widespread discrimination in Leipzig, as seen in other studies among refugee populations in Germany (Viazminsky et al., 2022). Discrimination is now recognized as a determinant of a range of health-related outcomes, including impaired acculturation and poor mental health (Borho et al., 2020; Şafak-Ayvazoğlu et al., 2021). This supports the need for programs that not only target individual psychological distress but also refugee community awareness programs that consider stigma, and also sensitization in wider Leipzig society to refugee mental health concerns. Prioritizing programs addressing professionals in health care, education, and the asylum administration would be a reasonable step due to their contact with refugee populations.

That over one-in-ten adults reported sexual assault and/or had another unwanted sexual experience is consistent with previous studies of sexual and gender-based violence (SGBV) among Syrians and other refugee populations (Davis, 2015; Freedman, 2016; UNHRC, 2018). This issue, which can be difficult to discuss, should always be considered in services that support this population (Asgary et al., 2013; Rizkalla et al., 2020).

### Limitations

We achieved only a low response rate to the postal survey, and this is common with a postal survey methodology. In addition to the adverts we placed, additional dialogs with key stakeholders and the use of social media could potentially have increased response rates. For future studies, social media will be increasingly important for community engagement but will have to be piloted to reflect the changing use of platforms, languages, and so on, and to take account of any differential impact by age, gender, and other characteristics that might reflect patterns of use. The low response rate obviously increases the risk of sampling bias. The external validity of our study sample (*N* = 513) closely reflected the sample frame for gender, with 61% men and 39% women in our final study sample versus 63.8% men and 36.2% women in the sample frame. For age, there was a little more variance: the proportion of respondents in our study sample (*N* = 513) versus the sample frame was: 39.0% versus 45.8% for age 18–30 years; 37.8% versus 36.89% for age 31–45 years, and 23.2% versus 17.31% for aged over 45 years. A potential bias associated with this could have been that those with mental health problems may have been more inclined to participate. Alternatively, someone with symptoms of depression/other mental health conditions might impair someone from completing the survey or having the motivation to participate. In addition, there was a relatively high number of respondents that were employed/student/retraining. This could introduce selection bias as they may have been more inclined to participate in our study due to greater literacy and trust in university institutions. The economic situation was self-assessed and so this subjective rating may not reflect objective data on income levels, although this is difficult to measure in populations living precarious lives. The sample size limited the statistical power of additional regression and effect modification analyses, especially in our ability to conduct disaggregated analysis (e.g., by gender). Related to this, the female gender was not associated with higher mental health needs, in contrast to some other studies with Syrian refugees (Hendrickx et al., 2020). In addition, other predictor variables could have been included in the regression modeling, such as discrimination.

Another possible limitation is that the sampling procedure of using two waves could have meant there were differences in sociodemographic variables between the two waves. However, there was no indication of this or that the time passed between the waves had any influence on participants’ mental health or sociodemographic variables. Our cross-sectional survey design did not allow us to explore mental health burden or access to services over time, recognizing that these are likely to fluctuate as refugee populations experience and adapt to changing circumstances, including acculturation and discrimination. We did not assess the quality of services that Syrian refugees receive and whether they were appropriate to their needs. Additionally, we only included officially registered Syrian refugees and those granted asylum in Germany as we had no data on unregistered or undocumented Syrian refugees, who we might expect to have greater mental health needs and even less access to services than our participants (Miller and Rasmussen, 2010). The use of single imputation in the regression analysis reduces statistical variance in the analysis, but missing data were low (Jakobsen et al., 2017). Finally, the study is limited to Leipzig and so cannot be generalizable to other cities in Germany.

## Conclusion

Our analysis of mental health symptoms and access to services by Syrian refugees in Leipzig, Germany found high rates of exposure to traumatic events, mental health distress, and discrimination. Using regression models, we found that both mental distress and somatization were associated with the use of services, in line with previous studies of Syrian refugees elsewhere. These findings reiterate the need for improved access to mental health care for the Syrian refugee community in Leipzig. Some recommendations are apparent. One is the need to ensure mental health services are more responsive to the needs of Syrian refugees in Leipzig, including greater use of translation services, greater awareness of refugees’ needs by health care providers – including somatic symptoms – and cultural competence. The delivery of scalable counseling and psychoeducation services by trained Syrian refugees could also be implemented more widely in Leipzig. Mental health awareness-raising programmes could also be scaled up within Syrian refugee communities in Leipzig. Finally, greater sensitization of the wider Leipzig society to Syrian refugee needs is required.

## Supporting information

Schoenberger et al. supplementary material Schoenberger et al. supplementary material

## Data Availability

Data will be made available. Please contact bayard.roberts@lshtm.ac.uk.

## Supplementary material

The supplementary material for this article can be found at https://doi.org/10.1017/gmh.2024.16.
