# Supplementary material for: Mental healthcare access among resettled Syrian refugees in Leipzig, Germany
Source: Glob Ment Health (Camb). 2024 Feb 6;11:e25. doi: 10.1017/gmh.2024.16 (PMC10988152; doi:10.1017/gmh.2024.16)
Supplement: Schoenberger et al. supplementary material [file S2054425124000165sup001.docx]

**TITLE: Mental healthcare access among resettled Syrian refugees in Leipzig, Germany**

SUPPLEMENTARY MATERIALS

**SUPPLEMENTARY MATEREIAL 1. Sample size calculation**

The aim is to achieve a total sample size of up to 500 men and women aged ≥18 years. This sample size was determined by the statistical requirements of a proposed descriptive and multivariate analysis of factors associated with mental health outcomes. To ensure adequate power to detect conceptually important differences within a multivariate analysis, the following parameters and calculations will be used: power = 80%; significance level = 5%; conceptually important difference in outcome scores = 0.8 SD (by convention a 'large' difference); size of 'rarest' sub-group of respondents we would attempt to include in our analysis = 5%; expected proportion of unusable questionnaires = 15%. To determine adequate sample size, the following calculation was made:

n = 2 x s^2^ x c

d^2^

n = number of people in one group

s = standard deviation

c = 7.85 (for 80% power and 5% significance level)

d = size of the difference to be detected (0.8 SD)

Using these parameters, a sample size of ~500 was required to detect a difference when only 5% of the population falls into a particular sub-group of interest.

**SUPPLEMENTARY MATERIAL 2. Questionnaire Measures**

*Discrimination*

Refugees were asked to report whether they “never,” “rarely,” or “often” experienced discrimination in a range of contexts, including at school or work, by public authorities, when looking for an apartment, and within the German health care system. For this analysis, “rarely” and “often” categories were collapsed to create a binary variable: “experienced discrimination” or “did not experience discrimination.”

*Traumatic events witnessed and experienced*

The DSM-5 Life Events Checklist (LEC-5) was used to assess trauma exposure (happened and witnessed) among participants. The LEC-5 considers the following types of events: physical and sexual assault, assault with a weapon, captivity and serious injury, or harm or death to someone else. A binary variable was also created for analyses, with the following options: “happened and/or witnessed” or “did not happen and/or witness.”

*Depression*

Depression was assessed with the Patient Health Questionaire-9 (PHQ-9), which contains nine items considering symptom presence in the previous two-week period. Participants rate each symptom on a Likert scale from 0 (“not at all”) to 3 (“nearly every day”). As validated in previous studies with the Arabic version of the PHQ-9, a summed PHQ-9 score was totaled for each participant and a binary variable created with a 10-point cut-off (“none/mild depression” vs. “moderate/severe depression”). Cronbach’s α in the current study sample was 0.89 (raw α 0.89).

*Anxiety*

The Generalized Anxiety Disorder Scale (GAD-7) considers the severity of generalized anxiety disorder symptoms with a seven-item self-report questionnaire. Severity is determined by a 4-point Likert scale: 0 (“not at all”) to 3 (“present nearly every day”) over a previous 2-week period. Scores were summed and a categorical variable created based on cut-offs previously used in studies of Syrian refugees: “≥ 5 mild”, “≥ 10 moderate (cut-off for clinical relevance)”, and “≥ 15 severe anxiety symptom levels”. Cronbach’s α in the current study sample was 0.91 (raw α 0.91).

*Post-traumatic stress disorder*

The Posttraumatic Stress Disorder Checklist for DSM-5 (PCL-5) assessed PTSD, as defined by DSM-5 criteria, with 20-items. Participants responded on a Likert-scale ranging from “not at all” to “extremely.” Total scale scores can range from 0-80. A binary “probable PTSD”/”no probable PTSD” variable was created using a ≥33 cut off, as indicated by previous studies with Syrian refugees^18^. Cronbach’s α in the current study sample was 0.95 (raw α 0.95).

*Current mental health symptoms*

A summary “current mental health symptomology” binary variable was created, composed of GAD-7, PHQ-9, and PCL-5. Participants who scored “moderate/severe depression,” and/or “probable PTSD,” and/or “moderate/severe” anxiety symptoms were coded as having current mental health symptoms. Those who scored “mild” or “none” on all three measures were coded as not having current mental health symptoms.

*Somatization*

The Somatic Symptom Scale-8 (SSS-8) assessed somatic symptom prevalence in the past week with a 5-point Likert scale ranging from 0 (“not at all”) to 4 (“very much”). Total scale scores can range from 0-32. Based on previous studies with Syrian refugees, a cut off score of >11 was used to create a binary variable for analysis with the following options: “somatization” vs. “no somatization”^14^. Cronbach’s α in the current study sample was 0.86 (raw α 0.86).

*Trust in Physicians*

The Wake Forest Trust in Physicians Scale, a 10-item questionnaire, assessed refugee trust in physicians. Respondents rated items on a 5-point Likert scale from 1 (“Strongly disagree”) to 5 (“Strongly agree”). Scores were summed, with higher values indicating greater trust in physicians. As no thresholds have been established for turning sum scores into a categorical variable, we reported mean and standard deviation for descriptive analyses. For regression and effect modification analyses, a binary variable was created based on the sample average to determine whether refugees had “above average trust in physicians” or “below average trust in physicians,” compared to others in the included sample. This scale has not been validated with Syrian refugees or Arabic-speaking populations. Cronbach’s α in the study sample was 0.87 (raw α 0.86).

**SUPPLEMENTARY MATERIALS 3. Poisson regression model building**

| **EXPONENTIATION OF CURRENT MENTAL HEALTH AND MENTAL HEALTHCARE** | | | | |
| --- | --- | --- | --- | --- |
| **Variable adjusted for** | **IRR** | **95% CI** | **p-value** | **AIC** |
| crude n=259 | 1.57 | 1.07 - 2.37 | **0.026** | 433.71 |
| sex n=259 (female as reference) | 1.55 | 1.05 - 2.34 | **0.032** |  |
| age n=259 (18-30 y/o as reference) | **1.65** | 1.12 - 2.50 | **0.014** |  |
| household econ (very good as dummy) n=244 | **1.48** | 1.00 - 2.26 | 0.058 |  |
| Education (basic as dummy), n=249 | 1.52 | 1.03 - 2.32 | **0.042** |  |
| Chronic condition (yes as reference) n=244 | 1.55 | 1.04- 2.37 | **0.037** |  |
| Arrival in Leipzig (before 2016 as reference) n=254 | 1.58 | 1.07 - 2.40 | **0.026** |  |
| adjusted model (age and household econ) n=244 | 1.54 | 1.03 - 2.36 | **0.039** | 414.92 |
| **EXPONENTIATION OF TRUST IN PHYSICIANS AND MENTAL HEALTHCARE ACCESS** | | | | |
| **Variable adjusted for** | **IRR** | **95% CI** | **p-value** | **AIC** |
| crude n=259 | 1.03 | 0.73 - 1.46 | 0.875 | 438.99 |
| sex n=259 (female as reference) | 1.03 | 0.73 - 1.47 | 0.861 |  |
| age n=259 (18-30 y/o as reference) | 1.06 | 0.75 - 1.52 | 0.740 |  |
| household econ (very good as dummy) n=244 | **0.99** | 0.69 - 1.42 | 0.946 |  |
| Education (basic as dummy), n=249 | **0.96** | 0.67 - 1.39 | 0.847 |  |
| Chronic condition (yes as reference) n=244 | 1.05 | 0.74 - 1.51 | 0.770 |  |
| Arrival in Leipzig (before 2016 as reference) n=254 | 1.02 | 0.71 - 1.46 | 0.932 |  |
| adjusted model (education and household econ) n=244 | 0.94 | 0.65 - 1.36 | 0.733 | 403.49 |
| **EXPONENTIATION OF SOMATIZATION AND MENTAL HEALTHCARE ACCESS** | | | | |
| **variable adjusted for** | **IRR** | **95% CI** | **p-value** | **AIC** |
| crude n=259 | 1.40 | 0.99 - 1.99 | 0.057 | 435.45 |
| sex n=259 (female as reference) | 1.38 | 0.97 - 1.96 | 0.073 |  |
| age n=259 (18-30 y/o as reference) | **1.46** | 1.02 - 2.07 | **0.036** |  |
| household econ (very good as dummy) n=244 | **1.36** | 0.95 - 1.96 | 0.094 |  |
| Education (basic as dummy), n=249 | 1.37 | 0.95 - 1.96 | 0.091 |  |
| Chronic condition (yes as reference) n=244 | 1.40 | 0.97 - 2.03 | 0.073 |  |
| Arrival in Leipzig (before 2016 as reference) n=254 | **1.43** | 1.00 - 2.03 | **0.049** |  |
| adjusted model (age, household economic situation and arrival in Leipzig) n=240 | 1.44 | 0.99- 2.10 | 0.055 | 408.19 |

**SUPPLEMENTARY MATERIALS 4. Effect modification of current mental health symptoms and mental healthcare service access by select third variables (n=259)**

Description:

Small cell sample size limited the ability to test for effect modification in this sample and contributed to large confidence intervals. While ORs differed for somatization when tested for effect modification (No current somatization OR: 1.62 95% CI: 0.86-3.05, current somatization OR: 5.03 95% CI: 0.96-26.34), cell sizes were too small and contributed to overlapping confidence intervals. While ORs differed for arrival in Leipzig when tested for effect modification (Arrival in Leipzig 2015 and earlier OR: 1.75 95% CI:0.81-3.78, Arrival in Leipzig 2016 to present OR: 2.96 95%CI: 1.42-6.17), cell sizes were too small and contributed to overlapping confidence intervals. While ORs differed for chronic condition status when tested for effect modification (no chronic condition OR: 1.80 95% CI: 0.94-3.45, chronic condition OR: 4.00 95% CI: 1.42-11.27), cell sizes were too small and contributed to overlapping confidence intervals. While ORs differed for years of education when tested for effect modification (Basic education OR: 6.97 95% CI: 1.59-30.52, Secondary education OR: 3.67 95% CI: 0.86-15.67, Post-secondary education OR: 1.50 95% CI: 0.78-2.86), cell sizes were too small and contributed to overlapping confidence intervals. Finally, while ORs differed for household economic situation when tested for effect modification (Good/very good OR: 3.50 95% CI: 0.76-16.12, Average OR: 1.69 95% CI: 0.85-3.33 Bad OR: 2.86 95% CI: 0.93-8.75), cell sizes were too small and contributed to overlapping confidence intervals.

There was no evidence for effect modification of trust in physicians on the relationship between current mental health symptoms and mental health care access (No trust in physicians OR: 2.36 95% CI: 1.06-5.29, Trust in physicians OR: 2.28 95% CI: 1.14-4.57), sex (Male OR: 2.26 95% CI: 1.17-4.34, Female OR: 2.23 95% CI: 0.92-5.43), and age group (18-30 y/o OR: 2.90 95% CI: 1.32-6.38, 31-45 y/o OR: 2.23 95% CI: 0.96-5.18, 46-95 y/o OR: 3.04 95% CI: 0.58-15.99). Again, cell sizes were too small and contributed to overlapping confidence intervals.

Supplementary Materials 4 Table:

| **CRUDE MODEL OF CURRENT MENTAL HEALTH AND MENTAL HEALTH CARE ACCESS** | | | |
| --- | --- | --- | --- |
|  | Sought care | Didn’t seek care | TOTAL |
| No current mental health concern | 59 | 33 | 92 |
| Current mental health concern | 73 | 94 | 167 |
| TOTAL | 132 | 127 | 259 |
|  | **PREVALENCE RATIO: 1.47 (1.17, 1.85)** | |  |
|  | **ODDS RATIO: 2.30 (1.36, 3.89)** | |  |
| **TRUST IN PHYSICIANS AS STRATIFIED VARIABLE** | | | |
|  | **For no trust in physicians** | |  |
|  | Did not seek care | Sought care | TOTAL |
| No current mental health concern | 25 | 13 | 38 |
| Current mental health concern | 35 | 43 | 78 |
| TOTAL | 60 | 56 | 116 |
|  | **PREVALENCE RATIO: 1.47 (1.05, 2.05)** | |  |
|  | **ODDS RATIO: 2.36 (1.06, 5.29)** | |  |
|  | **For trust in physicians** | |  |
|  | Did not seek care | Sought care | TOTAL |
| No current mental health concern | 34 | 20 | 54 |
| Current mental health concern | 38 | 51 | 89 |
| TOTAL | 72 | 71 | 143 |
|  | **PREVALENCE RATIO: 1.47 (1.08, 2.02)** | |  |
|  | **ODDS RATIO: 2.28 (1.14, 4.57)** | |  |
| **SSS8 AS STRATIFIED VARIABLE** | | | |
|  | **For no current somatization** | |  |
|  | Did not seek care | Sought care | TOTAL |
| No current mental health concern | 53 | 31 | 84 |
| Current mental health concern | 39 | 37 | 76 |
| TOTAL | 92 | 68 | 160 |
|  | **PREVALENCE RATIO: 1.23 (0.94, 1.62)** | |  |
|  | **ODDS RATIO: 1.62 (0.86, 3.05)** | |  |
|  | **For current somatization** | |  |
|  | Did not seek care | Sought care | TOTAL |
| No current mental health concern | 6 | 2 | 8 |
| Current mental health concern | 34 | 57 | 91 |
| TOTAL | 40 | 59 | 99 |
|  | **PREVALENCE RATIO: 2.01 (1.24, 3.25)** | |  |
|  | **ODDS RATIO: 5.03 (0.96, 26.34)** | |  |
| **SEX AS STRATIFIED VARIABLE** | | | |
|  | **For male** | |  |
|  | Did not seek care | Sought care | TOTAL |
| No current mental health concern | 42 | 21 | 63 |
| Current mental health concern | 47 | 53 | 100 |
| TOTAL | 89 | 74 | 163 |
|  | **PREVALENCE RATIO: 1.42 (1.08, 1.86)** | |  |
|  | **ODDS RATIO: 2.26 (1.17, 4.34)** | |  |
|  | **For female** | |  |
|  | Did not seek care | Sought care | TOTAL |
| No current mental health concern | 17 | 12 | 29 |
| Current mental health concern | 26 | 41 | 67 |
| TOTAL | 43 | 53 | 96 |
|  | **PREVALENCE RATIO: 1.51 (0.98, 2.32)** | |  |
|  | **ODDS RATIO: 2.23 (0.92, 5.43)** | |  |
| **AGE GROUP AS STRATIFIED VARIABLE** | | | |
|  | **For 18—30 years old** | |  |
|  | Did not seek care | Sought care | TOTAL |
| No current mental health concern | 29 | 16 | 45 |
| Current mental health concern | 25 | 40 | 65 |
| TOTAL | 54 | 56 | 110 |
|  | **PREVALENCE RATIO: 1.68 (1.15, 2.44)** | |  |
|  | **ODDS RATIO: 2.90 (1.32, 6.38)** | |  |
|  | **For 31—45 years old** | |  |
|  | Did not seek care | Sought care | TOTAL |
| No current mental health concern | 22 | 15 | 37 |
| Current mental health concern | 23 | 35 | 58 |
| TOTAL | 45 | 50 | 95 |
|  | **PREVALENCE RATIO: 1.50 (0.99, 2.27)** | |  |
|  | **ODDS RATIO: 2.23 (0.96, 5.18)** | |  |
|  | **For 46—95 years old** | |  |
|  | Did not seek care | Sought care | TOTAL |
| No current mental health concern | 8 | 2 | 10 |
| Current mental health concern | 25 | 19 | 44 |
| TOTAL | 33 | 21 | 54 |
|  | **PREVALENCE RATIO: 1.41 (0.94, 2.11)** | |  |
|  | **ODDS RATIO: 3.04 (0.58, 15.99)** | |  |
| **ARRIVAL CATEGORY AS EXPOSURE** | | | |
|  | **For 2015 and earlier** | |  |
|  | Did not seek care | Sought care | TOTAL |
| No current mental health concern | 23 | 16 | 39 |
| Current mental health concern | 37 | 45 | 82 |
| TOTAL | 60 | 61 | 121 |
|  | **PREVALENCE RATIO: 1.31 (0.92, 1.86)** | |  |
|  | **ODDS RATIO: 1.75 (0.81, 3.78)** | |  |
|  | **For 2016 to present** | |  |
|  | Did not seek care | Sought care | TOTAL |
| No current mental health concern | 36 | 16 | 52 |
| Current mental health concern | 35 | 46 | 81 |
| TOTAL | 71 | 62 | 133 |
|  | **PREVALENCE RATIO: 1.60 (1.18, 2.18)** | |  |
|  | **ODDS RATIO: 2.96 (1.42, 6.17)** | |  |
| **CHRONIC CONDITION AS EXPOSURE** | | | |
|  | **For no chronic condition** | |  |
|  | Did not seek care | Sought care | `TOTAL |
| No current mental health concern | 43 | 25 | 68 |
| Current mental health concern | 42 | 44 | 86 |
| TOTAL | 85 | 69 | 154 |
|  | **PREVALENCE RATIO: 1.29 (0.98, 1.72)** | |  |
|  | **ODDS RATIO: 1.80 (0.94, 3.45)** | |  |
|  | **For chronic condition** | |  |
|  | Did not seek care | Sought care | TOTAL |
| No current mental health concern | 14 | 7 | 21 |
| Current mental health concern | 23 | 46 | 69 |
| TOTAL | 37 | 53 | 90 |
|  | **PREVALENCE RATIO: 2.00 (1.27, 3.14)** | |  |
|  | **ODDS RATIO: 4.00 (1.42, 11.27)** | |  |
| **YEARS OF EDUCATION AS STRATIFIED VARIABLE** | | | |
|  | **For basic education** | |  |
|  | Did not seek care | Sought care | TOTAL |
| No current mental health concern | 10 | 3 | 13 |
| Current mental health concern | 11 | 23 | 34 |
| TOTAL | 21 | 26 | 47 |
|  | **PREVALENCE RATIO: 2.38 (1.34, 4.20)** | |  |
|  | **ODDS RATIO: 6.97 (1.59, 30.52)** | |  |
|  | **For secondary education** | |  |
|  | Did not seek care | Sought care | TOTAL |
| No current mental health concern | 11 | 3 | 14 |
| Current mental health concern | 16 | 16 | 32 |
| TOTAL | 27 | 19 | 46 |
|  | **PREVALENCE RATIO: 1.57 (1.01, 2.44)** | |  |
|  | **ODDS RATIO: 3.67 (0.86, 15.67)** | |  |
|  | **For post-secondary education** | |  |
|  | Did not seek care | Sought care | TOTAL |
| No current mental health concern | 35 | 26 | 61 |
| Current mental health concern | 45 | 50 | 95 |
| TOTAL | 80 | 76 | 156 |
|  | **PREVALENCE RATIO: 1.21 (0.89, 1.64)** | |  |
|  | **ODDS RATIO: 1.50 (0.78, 2.86)** | |  |
| **HOUSEHOLD ECON AS STRATIFIED VARIABLE** | | | |
|  | **For good/very good household economic status** | |  |
|  | Did not seek care | Sought care | TOTAL |
| No current mental health concern | 7 | 4 | 11 |
| Current mental health concern | 7 | 14 | 21 |
| TOTAL | 14 | 18 | 32 |
|  | **PREVALENCE RATIO: 1.91 (0.90, 4.05)** | |  |
|  | **ODDS RATIO: 3.50 (0.76, 16.12)** | |  |
|  | **For average household economic status** | |  |
|  | Did not seek care | Sought care | TOTAL |
| No current mental health concern | 40 | 22 | 62 |
| Current mental health concern | 41 | 38 | 79 |
| TOTAL | 81 | 60 | 141 |
|  | **PREVALENCE RATIO: 1.24 (0.94, 1.65)** | |  |
|  | **ODDS RATIO: 1.69 (0.85, 3.33)** | |  |
|  | **For bad household economic status** | |  |
|  | Did not seek care | Sought care | TOTAL |
| No current mental health concern | 10 | 7 | 17 |
| Current mental health concern | 18 | 36 | 54 |
| TOTAL | 28 | 43 | 71 |
|  | **PREVALENCE RATIO: 1.76 (1.02, 3.05)** | |  |
|  | **ODDS RATIO: 2.86 (0.93, 8.75)** | |  |
