# Supplementary material for: Mental healthcare access among resettled Syrian refugees in Leipzig, Germany
Source: Glob Ment Health (Camb). 2024 Feb 6;11:e25. doi: 10.1017/gmh.2024.16 (PMC10988152; doi:10.1017/gmh.2024.16)
Supplement: Peer review history

# Editor report: Recommendation: Mental healthcare access among resettled Syrian refugees in Leipzig, Germany — R0/PR2

**DOI:** 10.1017/gmh.2024.16.pr2

**Affiliations:** Program on Forced Migration and Health, Columbia University Mailman School of Public Health, United States

The reviewers recognized the importance of the article and were encouraging of its potential. They have made some recommendations and asked for several points of clarification, particularly in the methods and results sections. The reviewers also point out some areas of the discussion where the authors can elaborate on the interpretation and implications of the study findings. We hope you can consider these suggestions and are able to submit a revised draft of the manuscript.

# Editor report: Decision: Mental healthcare access among resettled Syrian refugees in Leipzig, Germany — R0/PR3

**DOI:** 10.1017/gmh.2024.16.pr3

**Affiliations:** Johns Hopkins University Bloomberg School of Public Health, United States

No accompanying comment.

# Editor report: Recommendation: Mental healthcare access among resettled Syrian refugees in Leipzig, Germany — R1/PR5

**DOI:** 10.1017/gmh.2024.16.pr5

**Affiliations:** Program on Forced Migration and Health, Columbia University Mailman School of Public Health, United States

Thank you for the thorough revision of this paper. I appreciate your thoughtful consideration of the sampling-related limitations. As mentioned by one of the reviewers, the low participation rate does raise some concerns, which you appropriately address in the limitations section of the discussion. However, given these concerns I ask that the authors qualify some of the interpretations in the results or earlier in the discussion section as well as in the abstract by more explicitly mentioning how and to whom these findings can (or can’t) be generalized. With these minor revisions, we agree that this paper is suitable and ready for publication. Thank you for considering this feedback.

# Editor report: Decision: Mental healthcare access among resettled Syrian refugees in Leipzig, Germany — R1/PR6

**DOI:** 10.1017/gmh.2024.16.pr6

**Affiliations:** Johns Hopkins University Bloomberg School of Public Health, United States

No accompanying comment.

# Editor report: Recommendation: Mental healthcare access among resettled Syrian refugees in Leipzig, Germany — R2/PR8

**DOI:** 10.1017/gmh.2024.16.pr8

**Affiliations:** Program on Forced Migration and Health, Columbia University Mailman School of Public Health, United States

No accompanying comment.

# Editor report: Decision: Mental healthcare access among resettled Syrian refugees in Leipzig, Germany — R2/PR9

**DOI:** 10.1017/gmh.2024.16.pr9

**Affiliations:** Johns Hopkins University Bloomberg School of Public Health, United States

No accompanying comment.
